# Directed evolution of G protein-coupled receptors in yeast for higher functional production in eukaryotic expression hosts

**DOI:** 10.1038/srep21508

**Published:** 2016-02-25

**Authors:** Marco Schütz, Jendrik Schöppe, Erik Sedlák, Matthias Hillenbrand, Gabriela Nagy-Davidescu, Janosch Ehrenmann, Christoph Klenk, Pascal Egloff, Lutz Kummer, Andreas Plückthun

**Affiliations:** 1 Department of Biochemistry, University of Zurich, Winterthurerstrasse 190, CH-8057 Zurich, Switzerland

## Abstract

Despite recent successes, many G protein-coupled receptors (GPCRs) remained refractory to detailed molecular studies due to insufficient production yields, even in the most sophisticated eukaryotic expression systems. Here we introduce a robust method employing directed evolution of GPCRs in yeast that allows fast and efficient generation of receptor variants which show strongly increased functional production levels in eukaryotic expression hosts. Shown by evolving three different receptors in this study, the method is widely applicable, even for GPCRs which are very difficult to express. The evolved variants showed up to a 26-fold increase of functional production in insect cells compared to the wild-type receptors. Next to the increased production, the obtained variants exhibited improved biophysical properties, while functional properties remained largely unaffected. Thus, the presented method broadens the portfolio of GPCRs accessible for detailed investigations. Interestingly, the functional production of GPCRs in yeast can be further increased by induced host adaptation.

With about 800 different members in humans, G protein-coupled receptors (GPCRs) are the largest superfamily of cell surface receptors^1^. Representing the most abundant transmitter of extracellular signals into the cell, GPCRs respond to a wide variety of ligands and transduce signals via different heterotrimeric G proteins as well as in a G protein-independent fashion^2^. The pivotal role of GPCRs is reflected by the great number of human diseases linked to aberrant GPCR signalling, including metabolic disorders, cardiovascular diseases, mental disorders, neurodegenerative diseases, and cancer^3^. As a consequence, GPCRs represent highly relevant drug targets, and about 30–50% of the currently marketed drugs act on these receptors^4^,^5^,^6^.

Much of our understanding about GPCRs has been obtained from the recent successes in structural investigations. However, the 30 unique GPCR structures deposited in the *Protein Data Bank* (PDB) to date still represent less than 4% of all receptors. Furthermore, the number of unique GPCR structures published per year has been constantly decreasing again during the last two years.

This trend reflects the intrinsic and yet unresolved limitations of structural investigations of GPCRs. The major challenges in such investigations are the difficulties to produce functional GPCRs in sufficient yields as well as the inherent receptor instability. Due to the establishment of integral membrane protein crystallization in lipidic cubic phases, which allows the use of mild detergents for receptor solubilization and brings the GPCR into a stabilizing, membrane-like environment of monoolein bilayers during crystallization, the instability of GPCRs has become less of a problem for structural biology^7^,^8^,^9^. However, the requirement to obtain sufficient functional material from heterologous expression still persists and often represents the main bottleneck for detailed investigations of GPCRs.

The strategy to produce GPCRs in sufficient yields has relied solely on using higher eukaryotic expression systems, for instance the baculovirus/insect cell system^10^. In fact, insect cells have been the most successful expression system. For about 85% of all GPCR structures deposited in the PDB, the receptors have been recombinantly produced in insect cells (not counting structures obtained from protein extracted from its natural source). However, the insect cell expression system does not provide a generic solution. Even in this production system, many GPCRs express at such low levels that, in order to obtain sufficient material, large-scale expression cultures would be required, which are either very costly or simply not feasible^11^,^12^,^13^.

Recently, our group addressed the problem of insufficient GPCR expression levels and we developed a system based on directed evolution to increase functional GPCR production in *Escherichia coli* by generating improved receptor variants^14^,^15^. Based on this method, several receptors have been evolved, and we were able to solve the structures of three different variants of the neurotensin receptor 1 (NTR1) which were expressed in *E. coli*^16^.

Despite the success of this approach, the system using *E. coli* has its limitations. For instance, to generate NTR1 variants suitable for crystallization, the variants obtained from the initial directed evolution approach required extensive further improvements in terms of expression levels and stability^17^,^18^,^19^,^20^. Furthermore, *E. coli* lacks the eukaryotic secretory quality control and translocation machinery required for the efficient production of many eukaryotic membrane proteins. Indeed, the expression of many wild-type GPCRs in *E. coli* is extremely toxic for the prokaryotic host. As a consequence, many GPCRs are not accessible for directed evolution in *E. coli*.

Therefore, we aimed to combine the advantages of the established GPCR evolution concept with the benefits of eukaryotic expression hosts into one integrative technology. Such a novel system should allow the directed evolution of a broad set of GPCRs by generating receptor variants with high expression levels in eukaryotic hosts. Thus, we hypothesized that it might be advantageous to perform the evolution of GPCRs directly in a eukaryotic host. For that purpose, the yeast *Saccharomyces cerevisiae* is a promising candidate. Due to its favourable characteristics of fast growth, cost-efficient cultivation, and ease of transformation, *S. cerevisiae* has been shown to be an ideal system for directed evolution, for instance in various applications of yeast surface display^21^,^22^,^23^,^24^. Despite being a comparatively simple eukaryotic organism, *S. cerevisiae* is equipped with the sophisticated cellular machinery of eukaryotes allowing efficient biosynthesis and translocation of complex membrane proteins, including two endogenously expressed GPCRs^25^. Therefore, yeast is also an attractive host for the recombinant production of membrane proteins. In fact, it has been shown that yeast tolerates recombinant GPCR expression quite well and that many different GPCRs can be expressed in *S. cerevisiae*, albeit usually at low levels^12^.

Here we introduce *SaBRE* (*Saccharomyces cerevisiae*-based receptor evolution), a fast, efficient, and robust method to increase the functional expression levels of GPCRs in eukaryotic hosts by directed evolution in yeast. With three different receptors, we show that the method is generally applicable, even for GPCRs which are very difficult to express in eukaryotic systems and are not accessible for directed evolution in *E. coli*. Our system allows the generation of receptor variants which show high functional expression in both yeast and insect cells and additionally exhibit improved biophysical properties. Importantly, the functional properties of the evolved GPCRs remain largely unaffected and similar to the wild-type receptors. In addition, we demonstrate that functional production of GPCRs in yeast can be further increased by induced host adaptation in a reproducible way.

## Results

### Two rounds of SaBRE are sufficient to obtain GPCR variants with highly increased expression levels in yeast

To demonstrate the general applicability of our method, we performed SaBRE with three different GPCRs.

We chose neurotensin receptor 1 (NTR1) and NK-1 receptor (NK1R; also known as tachykinin receptor 1 or substance-P receptor) to be subjected to SaBRE, since both of these receptors have been evolved in the *E. coli*-based system before^14^,^15^,^17^,^18^,^19^,^20^, allowing us to compare the variants obtained from evolution in *E. coli* with new SaBRE variants. As shown in several studies, NTR1 is readily evolvable in *E. coli*, and the obtained variants also expressed at increased levels in certain eukaryotic hosts^14^,^17^,^18^,^19^,^20^. In contrast, the evolution of NK1R in *E. coli* generated variants which show only moderate expression levels in the prokaryotic system^15^ and low expression levels in yeast (see below). This suggests that further improvement of functional NK1R expression may be possible, which would benefit future studies on this receptor.

For the third GPCR to be subjected to SaBRE, we chose the κ-type opioid receptor (KOR1) which, to our knowledge, has never been evolved before. Even in insect cells, KOR1 expresses at very low levels and thus represents a challenging example. Furthermore, the overexpression of KOR1 in *E. coli* results in extremely low functional production levels and high toxicity for the host (unpublished data). Therefore, KOR1 has been inaccessible for directed evolution so far.

**Figure 1:**
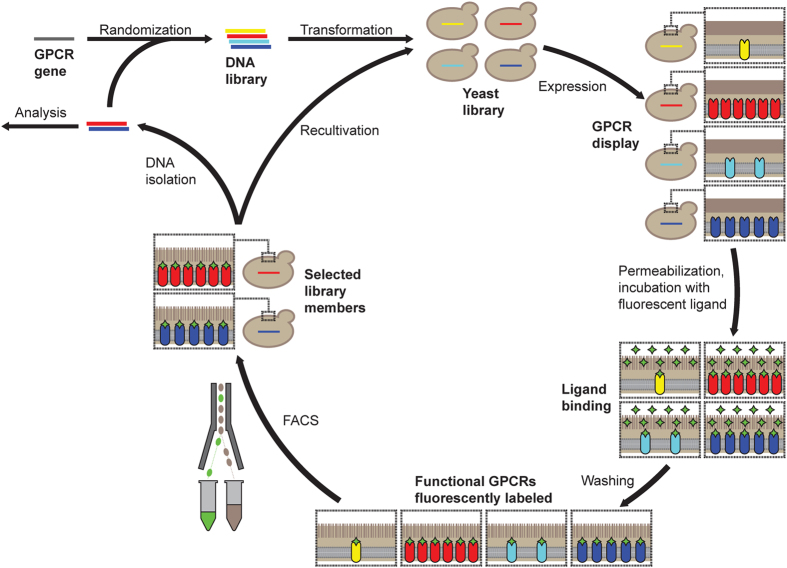
Workflow for directed evolution of GPCRs in yeast. Evolution of GPCRs starts with the generation of a DNA library by randomly mutagenizing the wild-type GPCR gene with error-prone PCR. Thereby, different variants with on average 2–5 non-silent mutations are created. The generated DNA library is then combined with the linearized expression vector and the mixture is used for transformation of yeast cells, during which the insert DNA and vector backbone are assembled *in vivo* by homologous recombination. The obtained yeast library comprises 5 × 10^7^ − 1 × 10^8^ different clones, each expressing a different GPCR variant (shown with different colors and different expression levels). After expression, the cells are permeabilized and incubated with fluorescent ligand (green diamonds) under saturating conditions. The fluorescent ligand binds exclusively to correctly folded GPCRs that are located in the plasma membrane, while unbound ligand is removed by washing. Correspondingly, cells producing receptor variants with high functional expression (here: red and dark blue) exhibit high fluorescence. Subsequently, these cells, which are expressing the desired GPCR phenotype, are selected during FACS by gating the top 0.5–1.0% of the most fluorescent cells. During FACS, the cells are directly sorted into growth medium for subsequent propagation. This selection by FACS is performed five times to obtain a strong enrichment of cells harbouring the best expressing GPCR variants. Whenever desired, the vectors coding for the GPCR variants can be isolated from the selected cells for analysis of individual mutants or for introduction of additional diversity by random mutagenesis for another round of evolution. Thus, one round of SaBRE includes one random mutagenesis followed by five selection rounds with FACS.

For each of the three chosen GPCRs, SaBRE was performed according to the same workflow (Fig. 1). First, the wild-type GPCR genes were randomized by error-prone PCR (typically 2–5 non-silent mutations in each generated variant), and the resulting DNA libraries were used for transformation of yeast cells. The generated yeast libraries had an average diversity of 5 × 10^7^ − 1 × 10^8^ different receptor variants. After expression, the yeast cells were treated with a buffer that was developed to permeabilize the cell wall by a reductive step in the presence of lithium acetate. This was necessary to allow diffusion of fluorescently labelled GPCR ligands through the cell wall. During the incubation with saturating concentrations of fluorescent ligands, only correctly folded GPCRs, which are located in the plasma membrane at the cell surface, are able to bind the ligand. Correspondingly, cells that express the desired phenotype – a GPCR variant which is produced at high functional levels – will bind more fluorescent ligand. After fluorescent ligand binding, unbound ligand was removed by washing and the cells were subjected to selections with fluorescence-activated cell sorting (FACS). During FACS, the most fluorescent cells were isolated and afterwards recultivated. In order to achieve a strong enrichment of those yeast cells that express the desired GPCR variants, this procedure of expression, fluorescent ligand binding, and FACS was performed five times in total. After the selection, the plasmids coding for the enriched variants were isolated from the selected library pools and subjected to a second round of evolution by re-randomization and FACS-based selection.

**Figure 2:**
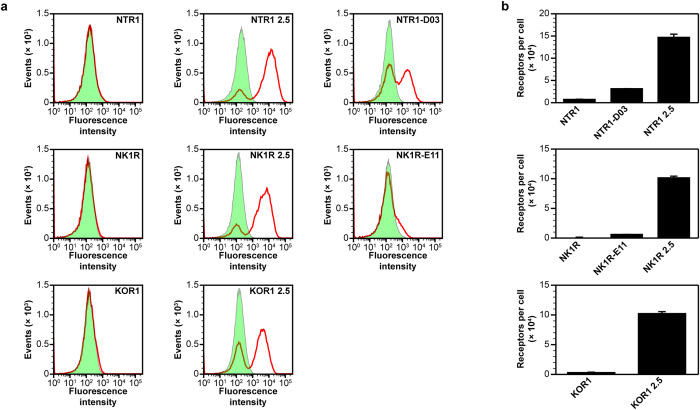
Expression levels in yeast after two rounds of SaBRE. (**a**) Histogram plots of fluorescent ligand-binding flow cytometry data of expressed NTR1, NK1R, and KOR1 variants. In these flow cytometry experiments, the amount of functional receptors at the surface of intact individual cells is determined. Compared is the functional surface expression level of wild-type GPCRs (left panels), library pools obtained after the two rounds of SaBRE (middle panel) and variants evolved in *E. coli* (right panels). The total signal (red curves) and the nonspecific signal (green, tinted) are shown. For the wild-type GPCRs (NTR1, NK1R, KOR1) no specific signal is obtained, thus no active receptor is detected at the surface. After two rounds of SaBRE, the selected library pools (NTR1 2.5, NK1R 2.5, KOR1 2.5) show a high specific signal, reflecting a high surface expression of functional GPCRs. Variants previously evolved in *E. coli* (NTR1-D03, NK1R-E11) show a specific signal as well, albeit at significantly lower levels than obtained for the SaBRE library pools and for a significant fraction of cells, no functional expression is detected at the surface (note the double peak of the total signal). For instance, only 50% of the cells express NTR1-D03 at the surface, while for NK1R-E11 only a minority of cells show active surface expression. (**b**) Measurement of average total functional GPCRs expressed per cell of NTR1, NK1R, and KOR1 variants by radioligand binding. In contrast to flow cytometry analysis, radioligand binding assays account for the total amount of functional receptors averaged across an entire population of lysed cells, and will thus detect functional GPCRs in intracellular membranes as well. The wild-type GPCRs show very low expression levels and the receptor variants previously evolved in *E. coli* show a low to moderate average functional production. In contrast, the selected SaBRE library pools show high functional expression levels with on average 100,000–150,000 receptors per cell, representing an increase of up to 50- and 20-fold, compared to the wild-type receptors and the variants previously evolved in *E. coli*, respectively. Error bars indicate standard deviations from triplicates.

For all three GPCRs we evolved, only two rounds of SaBRE were sufficient to strongly increase the functional expression levels in yeast (Fig. 2). The selected library pools (NTR1 2.5, NK1R 2.5, KOR1 2.5) showed up to a 50-fold increase in expression levels compared to the wild-type receptors. Furthermore, the expression levels of the selected libraries are also higher than the levels of variants previously evolved in *E. coli* from the same receptors (NTR1-D03, NK1R-E11), when those variants are expressed in yeast. Interestingly, the expression profiles measured by fluorescent ligand binding with flow cytometry revealed that the yeast cells are divided into two subpopulations, of which one subpopulation shows no surface expression of active receptor at all, while for the other subpopulation surface-expressed GPCR is detected. Since this division into two subpopulations was observed upon expression of all GPCRs under study in both single clones and libraries, this effect appears to be an intrinsic feature of GPCR expression in *S. cerevisiae*. Even though a loss of the plasmid in some cells cannot be excluded, our analysis revealed that plasmid-loss is not the sole reason for these bimodal expression profiles (*vide infra*).

In order to identify the individual GPCR variants generated with SaBRE, plasmid DNA was isolated from the selected library pools after the first and the second round of evolution and single clones were sequenced (Supplementary Fig. 1). Interestingly, whereas all selected NTR1 and KOR1 variants are full-length receptors, the obtained NK1R variants all contain a stop codon at different positions in the C-terminal part of the receptor, resulting in a shortened C-terminus, while all 7 transmembrane domains as well as the presumed helix 8 are always maintained. Since this represents a strongly selected feature for NK1R, it indicates that a shortened C-terminus is beneficial for expression of this receptor.

### GPCR variants generated with SaBRE show high functional expression in *Spodoptera frugiperda* (*Sf*9) insect cells

**Figure 3:**
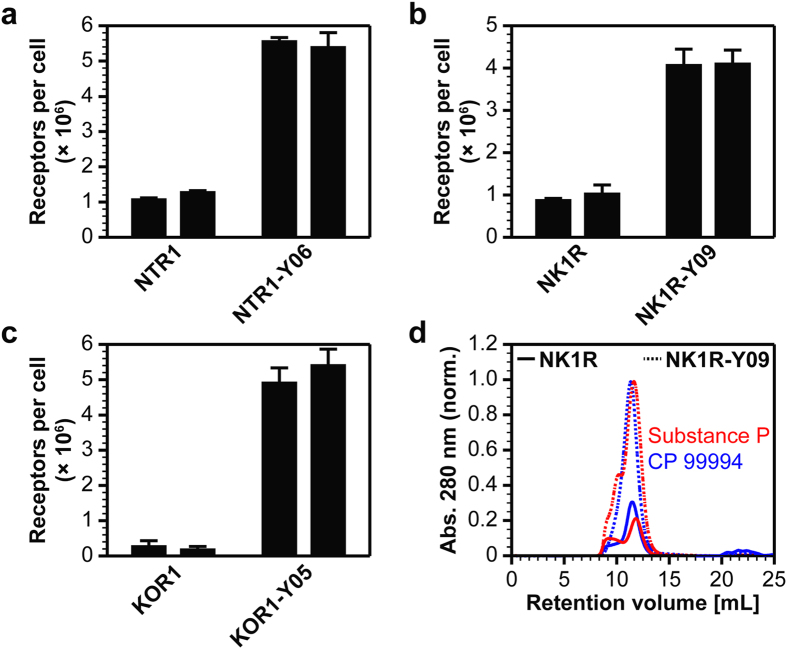
Expression of SaBRE variants in *Sf*9 insect cells and subsequent purification. (**a**–**c**) Measurement of average total functional GPCRs expressed per cell of NTR1, NK1R, and KOR1 variants by radioligand binding. Results from two independent expression experiments are shown (separate bars). The SaBRE variants show a significantly enhanced functional production in *Sf*9 insect cells with on average 4.0 × 10^6^ — 5.5 × 10^6^ functional receptors per cell. Compared to the corresponding wild-type receptors, NTR1-Y06 shows a 5-fold, NK1R-Y09 a 4-fold, and KOR1-Y05 a 26-fold increase in average functional expression. Error bars indicate standard deviations from triplicates. (**d**) Size-exclusion chromatography profiles of purified wild-type NK1R (solid lines) and NK1R-Y09 (dashed lines) purified in the presence of agonist (substance P, red lines) or antagonist (CP 99994, blue lines). Measured values for absorbance at 280 nm were normalized to the maximal absorbance obtained with NK1R-Y09. Equal amounts of cells were used for purification by immobilized metal ion affinity chromatography and the same volume of purified material was analyzed by size-exclusion chromatography. The size-exclusion chromatography profiles reflect the difference in functional expression levels and total yield of purified GPCR obtained with the wild-type receptor and the evolved variant. For NK1R-Y09, the yield of purified protein (3–6 mg/L) is increased by a factor of 4–5 compared to wild-type NK1R (≤1 mg/L).

Since we aimed to increase functional GPCR expression not only in yeast but in insect cells as well, we expressed the most enriched variants of each GPCR, NTR1-Y06 (10 mutations), NK1R-Y09 (8 mutations), and KOR1-Y05 (8 mutations), in *Sf*9 insect cells (Fig. 3a–c). Indeed, compared to the wild-type GPCRs, the evolved variants express at significantly higher levels in insect cells. For KOR1, notably the most challenging example, the evolved variant KOR1-Y05 shows a 26-fold increase in functional expression. Furthermore, the high expression levels of the SaBRE variants surpass the production levels of GPCR mutants evolved in the *E. coli*-based system, when those are expressed in *Sf*9 insect cells^18^.

For all GPCRs, expression was performed according to the same protocol. Thus, to obtain such high production levels of up to 5.5 × 10^6^ functional receptors per cell, no receptor-specific optimization is required, and expression cultures with a volume of about 1 L will give sufficient yields for detailed investigations. Indeed, we expressed and purified the NK1R variant NK1R-Y09 in the presence of agonist or antagonist (Fig. 3d). From such purifications, we reproducibly obtained 3–6 mg/L pure protein (Supplementary Fig. 2). Compared to the yield obtained for wild-type NK1R (≤1 mg/L), the increase in the purification yield is in good agreement with the higher expression levels of the evolved variant. Table 1 summarizes the expression levels obtained in *Sf*9 insect cells for the wild-type GPCRs as well as for the evolved variants.

### SaBRE also improves the biophysical properties of the GPCR variants, while the functional properties remain unaffected

**Figure 4:**
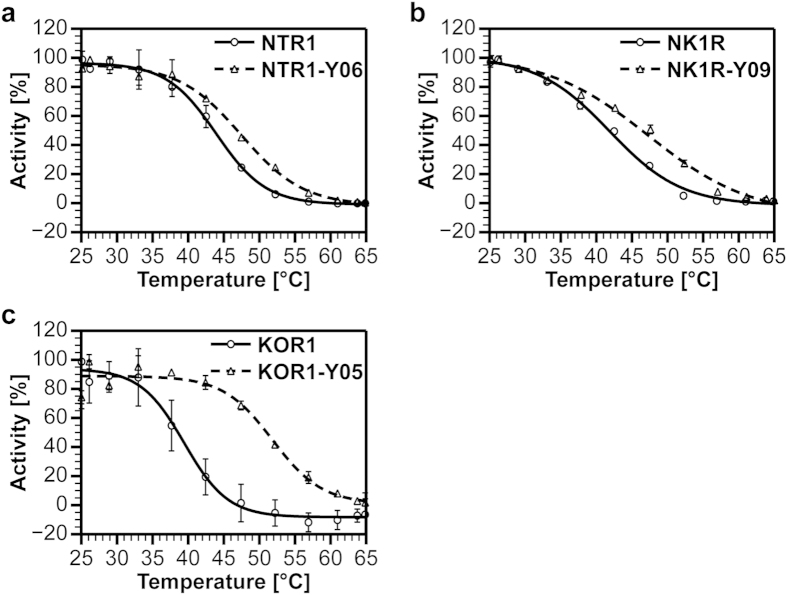
Thermostability measurements. Thermostability assays for wild-type receptors (solid lines, circles) and evolved SaBRE variants (dashed lines, triangles) measured with radioligand binding are shown. (**a**) Thermostability measurements of NTR1 and NTR1-Y06. Compared to wild-type NTR1, NTR1-Y06 shows higher thermostability with a relative increase of the melting temperature (ΔT_m_) of 3.6 ± 0.8 °C. (**b**) Thermostability measurements of NK1R and NK1R-Y09. Compared to wild-type NK1R, NK1R-Y09 shows a higher thermostability with a relative increase of ΔT_m_ of 5.4 ± 1.6 °C. (**c**) Thermostability measurements of KOR1 and KOR1-Y05. Compared to wild-type KOR1, KOR1-Y05 shows a higher thermostability with a relative increase of ΔT_m_ of 12.4 ± 1.7 °C. Error bars indicate standard deviations from duplicates.

In previous studies, it has been shown that evolved GPCR variants with improved functional expression also frequently exhibit a higher stability^14^,^15^,^18^. Since this is a very advantageous effect of directed evolution towards higher functional expression, which may benefit purification yields, crystallization attempts, as well as *in vitro* studies, we performed thermostability measurements with the wild-type receptors and the evolved variants to investigate whether also SaBRE leads to such an improvement of the biophysical properties (Fig. 4). For each of the evolved GPCRs, we measured increased values for the melting temperatures (T_m_) in *Sf*9 membranes compared to the T_m_ values obtained for the wild-type receptors (summarized in Table 1). Thus, the SaBRE variants show indeed a higher thermostability. Interestingly, the highest relative increase in thermostability (ΔT_m_) of about 12 °C is observed for KOR1-Y05 (Fig. 4c), which also shows the highest relative increase in functional expression (Fig. 3c). This indicates that the increased stability of the receptor variants may be one of the main factors contributing to the improved expression of the obtained SaBRE variants.

**Figure 5:**
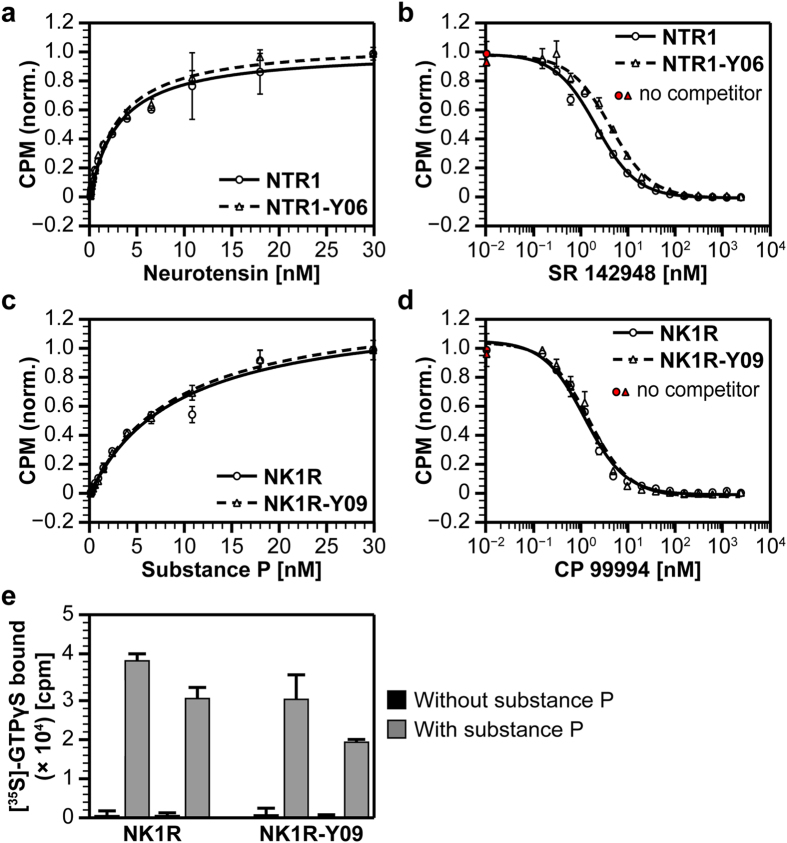
Analysis of the functional properties of the evolved GPCR variants. (**a**,**c**) Determination of the apparent binding affinity of wild-type receptors (solid lines, circles) and evolved SaBRE variants (dashed lines, triangles) for agonists (neurotensin and substance P) by radioligand saturation binding. The apparent binding affinities (K_d_^app^ ) of the corresponding receptors for neurotensin or substance P remain unaltered for the evolved receptors compared to the wild-type GPCRs. Neurotensin is bound by NTR1 with K_d_^app^ = 3.0 ± 0.4 nM and by NTR1-Y06 with K_d_^app^ = 3.0 ± 0.3 nM. Substance P is bound by NK1R with K_d_^app^ = 9.4 ± 1.6 nM and by NK1R-Y09 with K_d_^app^ = 9.2 ± 0.7 nM. Error bars indicate standard deviations from duplicates. (**b**,**d**) Determination of the apparent binding affinity of wild-type receptors (solid lines, circles) and evolved SaBRE variants (dashed lines, triangles) for antagonists (SR 142948 and CP 99994) by radioligand competition binding. The apparent binding affinities of the corresponding receptors for SR 142948 and CP 99994 remain unaltered or very similar for the evolved receptors compared to the wild-type GPCRs. SR 142948 is bound by NTR1 with K_d_^app^ = 1.2 ± 1.1 nM and by NTR1-Y06 with K_d_^app^ = 2.3 ± 1.1 nM. CP 99994 is bound by NK1R with K_d_^app^ = 1.0 ± 1.1 nM and by NK1R-Y09 with K_d_^app^ = 1.2 ± 1.1 nM. Error bars indicate standard deviations from duplicates. (**e**) Measurement of signalling activity of NK1R variants by [^35^S]-GTPγS binding. Equal amounts of active GPCR were assayed with identical concentrations of purified and reconstituted G protein in the absence (black) and presence (grey) of the agonist substance P. Results of two independent signalling assays performed with two independent GPCR expressions are shown (separate bars). The wild-type receptor as well as the evolved variant show low basal activity without agonist stimulation. Upon addition of substance P, signalling is detected by [^35^S]-GTPγS binding which for NK1R-Y09 remains similar to the wild-type receptor. Error bars indicate standard deviations from triplicates.

The improved expression and stability clearly supports the use of GPCR variants evolved by SaBRE in future studies. However, it is of great importance that the obtained GPCR mutants still have functional properties similar to the wild-type receptors. Therefore, we determined the apparent dissociation constants for different ligands of the NTR1 and NK1R SaBRE variants and the corresponding wild-type GPCRs (Fig. 5a–d). The affinities for the agonists neurotensin (NTR1) and substance P (NK1R), which were used as fluorescently labelled versions during SaBRE, remain identical to the values measured for the wild-type receptors for both evolved GPCR variants. Strikingly, this holds also true for the small-molecule antagonists SR 142948 (NTR1) and CP 99994 (NK1R), both of which were not used during any step of the evolution. Thus, ligand affinities remain largely unaltered by SaBRE. The determined apparent dissociation constants for the different ligands of both GPCRs are summarized in Table 1.

Naturally, GPCR functionality cannot exclusively be assayed by ligand binding experiments, which represents only the first step of GPCR stimulation. A functional GPCR needs to be able to activate the heterotrimeric G proteins. As no high-resolution structure of NK1R is available so far, well-expressed and stable mutants of this receptor are of particular interest. Since NK1R-Y09 can be readily purified at high yields (Fig. 3d), this evolved variant represents the most interesting candidate for further characterization. Therefore, we analyzed the signalling activity of NK1R-Y09 in comparison with wild-type NK1R by performing *in vitro* signalling assays based on binding of [^35^S]-GTPγS with purified heterotrimeric G protein (Fig. 5e). The evolved variant shows similar signalling activity as the wild-type receptor, both regarding basal activity without stimulation and upon stimulation with the agonist substance P. This illustrates that SaBRE variants can be biologically active and able to perform their naturally intended function.

### Yeast clones can be reproducibly adapted towards higher functional GPCR production

**Figure 6:**
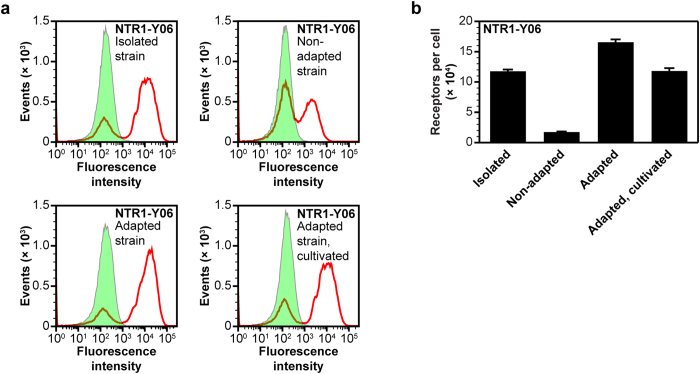
Analysis of induced adaptation in yeast cells expressing the evolved receptor variant NTR1-Y06. (**a**) Expression profiles of different yeast strains expressing NTR1-Y06 measured by ligand binding flow cytometry experiments. Compared is the functional surface expression of NTR1-Y06 in a yeast strain isolated from the selected library pool NTR1 2.5, in a freshly transformed non-adapted strain, in a strain adapted by repetitive selection with FACS, and in an adapted strain that has been repetitively cultivated under non-expressing conditions. The total signal (red curves) and the nonspecific signal (green, tinted) are shown. The isolated strain shows a similar expression profile as detected for the NTR1 2.5 library pool (cf. Fig. 2a). In contrast, the freshly transformed and non-adapted strain shows a lower specific signal, corresponding to a decreased surface expression of NTR1-Y06. Furthermore, the subpopulation of cells showing no surface expression at all is increased in the non-adapted strain (note the increase of the left peak of the total signal double peak compared to the isolated strain). By five repetitive selections with FACS, the freshly transformed yeast strain can be adapted, which leads again to the high-expression profile. If the adapted strain is repetitively cultivated prior to induction of expression under non-expressing conditions, the average expression level decreases again, depicted by a drop of the specific signal in combination with an increase of the fraction of cells with no surface expression of NTR1-Y06. (**b**) Measurement of average total functional GPCRs produced per cell by radioligand binding. The data show the significant difference in the average number of active NTR1-Y06 receptors per cells between non-adapted and adapted strains. As shown by the flow cytometry data, the higher total expression levels can be explained by a combined effect of an increased surface expression per cell in the expressing subpopulation of cells and a decrease of the fraction of cells showing no active surface expression. Upon repetitive cultivation of the adapted strain under non-expressing conditions, the average functional receptor levels drop by about 30%. Error bars indicate standard deviations from triplicates.

In addition to analysis in insect cells, we also performed expression experiments of the SaBRE variants in yeast. We noticed that the functional expression dropped when fresh yeast cells were retransformed with plasmids encoding evolved GPCRs. In contrast, a single clone expressing NTR1-Y06 isolated from the selected library pool NTR1 2.5 showed the expected high expression profile (Fig. 6).

We hypothesized that during the repetitive selection with FACS in SaBRE, a cellular adaptation of the clones occurred, which lead to additional improvements of the expression levels. Indeed, we were able to induce this effect by five repetitive selections with FACS in a freshly transformed yeast clone which expresses the evolved receptor NTR1-Y06 (Fig. 6). During this phenotypic selection without mutagenesis, the adaptive effect gradually increased towards the expected high-expression profile (Supplementary Fig. 3). Conversely, by repetitive cultivation of the adapted strain under non-expressing conditions, we observed a partial reversion of the adaptive effect by a 30% drop of the expression level (Fig. 6).

This adaptation is not receptor-specific, since yeast clones expressing NK1R-Y09 and KOR1-Y05 have also been adapted (Supplementary Fig. 4). Attempts to induce this adaptation in clones expressing wild-type NTR1 failed (data not shown). Thus, adaptation appears to be only possible if the expression of the protein of interest is well tolerated by the cells, which is the case for our evolved variants but not for many wild-type GPCRs.

**Figure 7:**
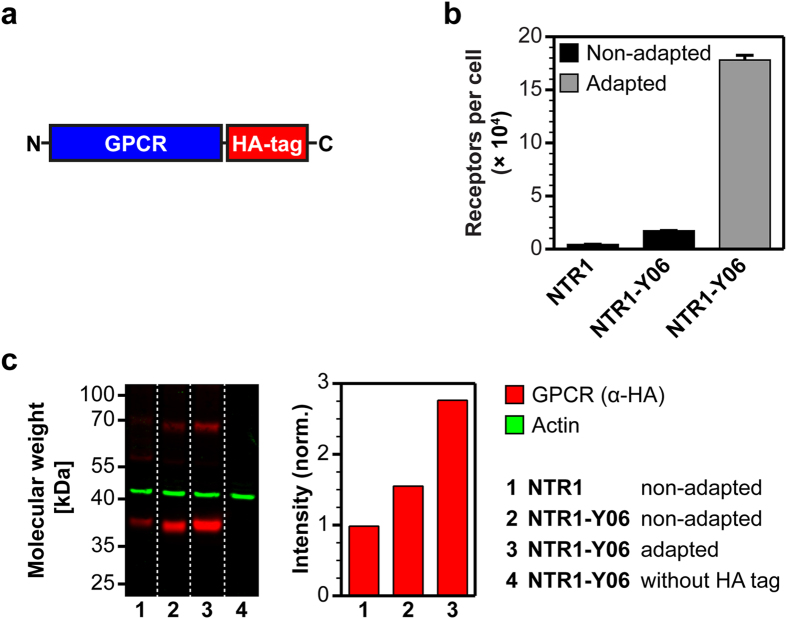
Quantification of total GPCR produced in non-adapted and adapted yeast strains. (**a**) GPCR expression construct with a C-terminal HA-tag used for the quantification of the total amount of receptor produced. (**b**) Measurement of average total functional GPCRs produced per cell of HA-tagged NTR1 variants expressed in non-adapted and adapted yeast strains by radioligand binding. Compared to expression of HA-tagged NTR1-Y06 in the non-adapted strain, adaptation leads to a further increase in average total functional production by a factor of 10. Error bars indicate standard deviations from triplicates. (**c**) Quantitative Western blot analysis of HA-tagged NTR1 variants expressed in non-adapted and adapted yeast strains. Equal numbers of cells were lysed for protein extraction and actin was used as a loading control (green). GPCRs were detected via their HA-tag (red), with main bands corresponding to monomeric GPCRs and bands of higher molecular weight, most likely representing GPCR dimers not disintegrated under the conditions used. For quantification (bar chart), intensities of all defined bands were accounted for. In the non-adapted strains, the total GPCR produced increases approximately 1.5-fold from wild-type NTR1 (lane 1) to NTR1-Y06 (lane 2). While the total amount of NTR1-Y06 produced increases also slightly when expressed in the adapted strain (lane 3) compared to expression in the non-adapted strain, the relative increase of total receptor produced (approximately 1.8-fold) is much lower than the increase in functional receptor observed in radioligand binding (approximately 10-fold). For a negative control (lane 4), cells expressing NTR1-Y06 without a HA-tag were used.

**Figure 8:**
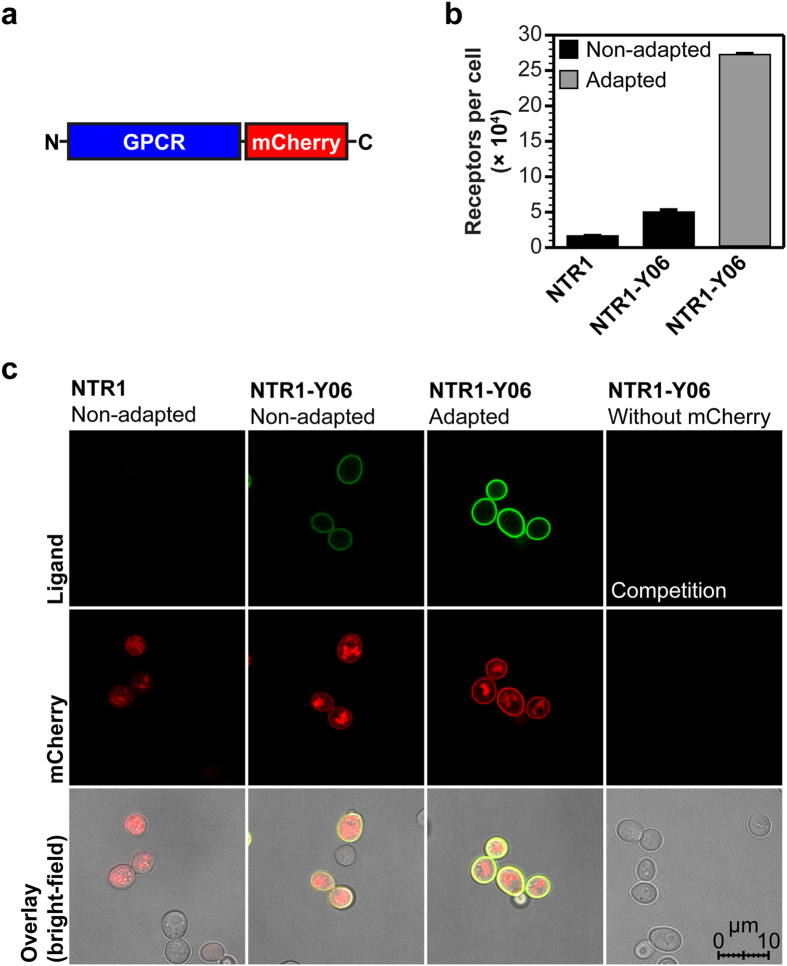
Analysis of intracellular and surface-expressed GPCR in non-adapted and adapted yeast strains. (**a**) GPCR expression construct with a C-terminal fusion to mCherry used for the detection of intracellular and surface-expressed receptor. (**b**) Radioligand binding measurements of average total functional GPCRs produced per cell of NTR1 variants with a C-terminal fusion to mCherry expressed in non-adapted and adapted yeast strains. Compared to expression of the NTR1-Y06-mCherry fusion in the non-adapted strain, adaptation leads to a further increase in average total functional production by a factor of 6. Error bars indicate standard deviations from triplicates. (**c**) Confocal fluorescence microscopy studies of NTR1 variants with a C-terminal fusion to mCherry in non-adapted and adapted yeast strains. Fluorescence intensities obtained by fluorescent ligand binding (top row, green) or from mCherry (middle row, red) as well as bright-field microscopy overlays (bottom row) are shown. For expression of wild-type NTR1 in the non-adapted strain (first column), no ligand binding signal at the cell surface is detected. A distinct mCherry signal is exclusively located in the cell interior, reflecting intracellularly retained receptor, which is mostly inactive according to the radioligand binding data. For expression of NTR1-Y06 in the non-adapted strain (second column), functional receptor at the surface is detected by fluorescent ligand binding. Similar as for expression of wild-type NTR1, the detected signal for mCherry is still localized to a large extent in the cell interior. For expression of NTR1-Y06 in the adapted strain (third column), strong signals for both fluorescent ligand binding and mCherry are observed at the surface, with only little mCherry detected in the cell interior. For a negative control (fourth column), cells expressing NTR1-Y06 without a mCherry fusion were incubated with fluorescently labelled ligand in excess of non-labelled ligand. Representative pictures are shown.

In order to analyze this adaptation, we created NTR1 and NTR1-Y06 versions with a C-terminal hemagglutinin-tag (HA-tag) (Fig. 7a) or a fusion to mCherry (Fig. 8a) and adapted yeast cells expressing the evolved variants. The HA-tagged GPCR variants allowed us to quantify the total amount of receptor produced in non-adapted and adapted strains by quantitative Western blot analysis and the mCherry fusions were used for protein localization studies by confocal microscopy to assess the amount of intracellular receptor.

By measuring the expression levels of the HA-tagged GPCRs in the non-adapted and the adapted strain, we observed that the *functional* expression increased from the non-adapted to the adapted strain by a factor of 10 (Fig. 7b). In contrast, the *total* amount of produced receptor measured by quantitative Western blot was very similar between the two strains (Fig. 7c). This indicates that the fraction of active receptor is significantly increased in the adapted strains.

With confocal microscopy, we observed that the increase of functional expression in the adapted strain (Fig. 8b) correlates with a higher fraction of receptor at the surface and, consequently, with a decreased amount of intracellularly retained receptor (Fig. 8c). According to the radioligand binding data (Fig. 8b), which account for the total amount of functional receptor including any putative active fraction of intracellular receptors, most of the intracellularly retained GPCR molecules in the non-adapted strains must be inactive. To further confirm the qualitative results from confocal microscopy, we performed flow cytometry analysis of the GPCR-mCherry fusions (Supplementary Fig. 5). Analysis of the mCherry signal revealed that the population of cells showing no active surface expression of GPCR consists of two further subpopulations, of which one subpopulation shows no expression at all (possibly cells that may have lost the plasmid), while the other one exclusively expresses intracellularly retained receptor. In the adapted strains, both of these subpopulations are decreased, leading to the overall higher functional expression levels. Furthermore, the weak correlation of the fluorescent ligand binding signal (functional receptor at the surface) to the mCherry signal (total receptor produced) in non-adapted strains and especially for the expression of wild-type NTR1 clearly supports the observation from confocal microscopy that a substantial amount of the produced receptor is intracellularly retained. Moreover, the finding that the fluorescence intensity of GPCRs genetically fused to a fluorescent protein correlates only weakly with functional receptor levels has been described before^26^,^27^.

## Discussion

During the last years, structural investigations of GPCRs have been advanced by the establishment of new crystallization methods. However, due to low functional expression yields, many GPCRs still remain refractory to such studies. We addressed this problem and were able to establish a simple and robust directed evolution method, called SaBRE, that efficiently allows improving the functional GPCR production in both yeast and insect cells.

The obtained GPCR variants expressed in insect cells at such high levels that expression cultures with a volume of about 1 L are sufficient to obtain the yields required for crystallographic or biochemical investigations. Remarkably, these SaBRE variants were generated in only two rounds of evolution, which can be easily done within 7 weeks, and several GPCRs can be evolved in parallel. Thus, SaBRE is considerably faster than a previously established directed evolution method using *E. coli*, for which usually more rounds of evolution were required^14^,^15^. Moreover, the obtained SaBRE variants showed higher functional expression in eukaryotic hosts than the corresponding variants generated in the *E. coli*-based system^18^.

A further advantage of SaBRE is the broader set of GPCRs which can be evolved in this system. For instance, expression of KOR1 in *E. coli* results in such low functional yields and high toxicity that evolution of KOR1 is hardly possible in that system. Since KOR1 has been successfully evolved in yeast, SaBRE represents a significant advancement of directed evolution methods, allowing more receptors to be evolved, and thus improving the perspectives for detailed investigations of more GPCRs.

Indeed, next to the three receptors presented in this study, SaBRE has been successfully applied to two additional GPCRs, namely the oxytocin and the parathyroid hormone 1 receptor (unpublished data), which further demonstrates the general utility of the method. Notably, the recombinant expression of both the oxytocin and the parathyroid hormone 1 receptor is extremely difficult, resulting even in insect cells in very low yields. With SaBRE, we generated variants of both of these receptors that show significantly increased expression levels. Similar as for KOR1, this would not have been possible with the *E. coli*-based directed evolution method.

Further characterization of the obtained SaBRE variants revealed that the generated receptor variants had also improved biophysical properties compared to their wild-type counterparts, which may further benefit the purification and, potentially, the crystallization of these variants. Since it has been observed before that evolution towards higher expression also generates GPCR variants of higher stability^14^,^15^,^18^, the increased stability is most likely an important factor contributing to higher functional expression. This is further supported by the fact that the highest relative increase in stability was detected for KOR1-Y05, for which also the highest relative increase in expression was measured.

Importantly, the functional properties of the generated SaBRE variants remained largely unaffected. Determination of ligand affinities revealed that the ligands used during the selections as well as ligands which were not used for the receptor evolution are bound by the evolved variants with identical apparent dissociation constants compared to the wild-type receptors. Furthermore, SaBRE can generate variants which are signalling-active, as demonstrated with one NK1R variant.

While performing SaBRE, we further observed an interesting effect on the yeast host cell. We were able to reproducibly induce adaptation of yeast cells towards higher functional GPCR production by repetitive selections with FACS. However, the prerequisite is that the yeast clone to be adapted expresses a receptor whose expression is not too toxic for the cell, for instance an evolved receptor variant. Attempts to induce the adaptation in cells expressing a wild-type GPCR failed.

Adapted yeast strains show a significant increase of surface-expressed receptor, leading to a much higher fraction of functional receptors, with only a small increase of the total amount of receptor produced. In contrast, in non-adapted strains, a large amount of receptors remain in intracellular compartments, of which a significant fraction represents inactive protein. The higher fraction of active receptors in combination with an increase of surface expression and a decrease of the non-expressing cell subpopulation leads to the overall higher average functional production yields in expression cultures of adapted yeast cells.

Since the yeast cells were never subjected to any mutagenic conditions and the adaptive effect can be partially reverted, we exclude that mutations, which could lead to such an effect, were acquired and selected in the host genome. We rather suspect that specifically regulated host cell responses are responsible for adaptation. Thereby, GPCR production is better tolerated in adapted strains and stress responses, like the unfolded protein response in the endoplasmic reticulum, which are typically induced upon GPCR expression and correlate with low functional yields^26^,^28^, may be decreased.

Clearly, to elucidate the exact nature of the described adaptive effect, further studies are required, which were beyond the scope of this work. Nevertheless, a profound understanding of the adaptive effect might open the door for rational strain engineering of *S. cerevisiae*^29^,^30^ or even for other hosts. Furthermore, the fact that expression of evolved GPCRs in yeast can be further improved by induced adaptation beyond the effect of the mutations themselves implies a potential of yeast as a large-scale production host. However, it remains to be tested, whether the concept of expression of improved receptor variants in combination with host adaptation can be applied to other yeast species as well, for instance to the commonly used protein production host *Pichia pastoris*. Since isotope-labelling of proteins is well established in *P. pastoris*^31^, this would especially benefit NMR studies.

## Methods

### Yeast expression

For all experiments the *S. cerevisiae* strain BY4741 (*MAT***a** *his3*∆*1 leu2*∆*0 met15*∆*0 ura3*∆*0*)^32^, obtained from EUROSCARF, was used. Standard expression was performed with pMS03het, derived from p415 GAL1^33^. To obtain pMS03het, the α-mating factor prepro sequence was cloned from pPICZα A (Life Technologies) into the multiple cloning site (*Xho*I/*Spe*I) of p415 GAL1. pMS03het contains *Nhe*I/*Bam*HI restriction sites which allow efficient vector linearization for high-efficiency transformation or in-frame cloning of genes preceded by the α-mating factor prepro sequence. For expression of GPCRs with a C-terminal HA-tag or fusion to mCherry, vectors pMS03het_HA or pMS03het_mCh were used, respectively. To obtain pMS03het_HA and pMS03het_mCh, sequences coding for the HA-tag or mCherry were cloned via *Bam*HI into pMS03het.

BY4741 cells transformed with pMS03het vectors were cultivated at 30 °C in SDD-Leu^–^ medium (6.9 g/L yeast nitrogen base without amino acids (Formedium), 690 mg/L complete supplement mixture without leucine (Formedium), 20 g/L glucose, 35 mM sodium citrate tribasic, 35 mM citric acid). For expression, yeast cells in the logarithmic growth phase grown in SDD-Leu^–^ medium at 30 °C were centrifuged and subsequently resuspended in SDG-Leu^–^ medium (identical to SDD-Leu^–^ but with 20 g/L galactose instead of glucose). Initial OD_600_ was always chosen to be 1.0 after resuspension in SDG-Leu^–^ and expression was performed at 20 °C for 24 h.

### DNA library construction and yeast transformation

The wild-type gene of rat NTR1 (N-terminally truncated from amino acids 1–42) was a kind gift from Reinhard Grisshammer (National Institutes of Health). Wild-type cDNA of human NK1R and human KOR1 was obtained from the Missouri S&T cDNA Resource Center. All wild-type GPCR genes were cloned into pMS03het (*Nhe*I/*Bam*HI).

DNA library construction was performed by amplification of wild-type genes or isolated DNA after the first round of evolution with error-prone PCR using the GeneMorph II random mutagenesis kit (Agilent Technologies) according to the manufacturer’s protocol. In each case, two error-prone PCRs (epPCRs) were performed, one epPCR with 20 and one with 25 cycles, of which the obtained products were subsequently pooled. Primers used for epPCR (forward primer: 5′–CTAAAGAAGAAGGGGTATCTCTCGAGAAACGTGAGGCGGAAGCGGCTAGC–3′; reverse primer: 5′–ATTACATGACTCGACTCGATGCCGACGAGAGCGGCCGCCTATTAGGATCC–3′) introduced sites homologous to linearized pMS03het (digested with *Nhe*I/*Bam*HI) at each end of the gene, allowing *in vivo* vector assembly by homologous recombination after co-transformation of PCR fragments with linearized pMS03het. Purified epPCR products were further amplified by standard PCR with the identical primers in order to obtain enough DNA for transformation.

High-efficiency transformation of BY4741 with DNA libraries was performed by square wave electroporation on a GenePulser Xcell electroporator (Bio-Rad) according to a previously published method^34^. Yeast cells were grown in 60 mL YPD at 30 °C to an OD_600_ = 1.8–2.0. As soon as this cell density was reached, 50 mL of culture were centrifuged, the medium aspirated, and cells were treated in 25 mL conditioning solution (100 mM lithium acetate, 10 mM DTT) at 30 °C for 15 min. Subsequently, cells were pelleted, washed in 25 mL cold ddH_2_O, pelleted again, and resuspended in cold ddH_2_O to a total volume of 500 μL. Henceforward, cells were always kept at 4 °C. For one transformation, 250 μL of yeast cells were mixed with 4 μg of linearized pMS03het and 12 μg PCR product and the transformation mixture was transferred to a 2 mm electroporation cuvette. Square wave electroporation was performed with one pulse with a voltage of 500 V and a pulse length of 15 ms. After electroporation, cells were allowed to recover in 5 mL YPD without shaking at 30 °C for 1 h. Finally, recovered cells were pelleted, transferred to 500 mL SDD-Leu^–^ for selective growth at 30 °C for 20–24 h, and stored in glycerol stocks at −80 °C. To obtain high-diversity libraries, always two transformations per library were performed. On average, libraries with a diversity of 5 × 10^7^ − 1 × 10^8^ were obtained.

### Fluorescent ligand binding with yeast cells

All fluorescent ligands were obtained by labelling with HiLyte Fluor 488 (AnaSpec). Neurotensin (8–13) (KKPYIL) was covalently labelled at the N-terminal amino group, substance P (RPKPQQFFGLM) was covalently labelled at the amino group of K^3^, and dynorphin A (1–11) (YGGFLRRIRPK) was covalently labelled at the amino group of K^11^.

In order to permeabilize the yeast cells for fluorescent ligand binding, expression cultures were centrifuged, medium was aspirated, and cells were resuspended in TELi buffer (50 mM Tris-HCl pH 9.0 (at 4 °C), 1 mM EDTA, 100 mM lithium acetate) at RT. Next, cells were incubated in TELi Buffer supplemented with 50 mM DTT at 20 °C for 30 min and subsequently washed twice in cold TELi Buffer. Henceforward, cells were always kept at 4 °C.

For fluorescent ligand binding, permeabilized cells were incubated with fluorescently labelled ligand (NTR1 variants: 25 nM fluorescent neurotensin (8–13); NK1R variants: 20 nM fluorescent substance P; KOR1 variants: 10 nM fluorescent dynorphin A (1–11)) in TELi buffer at 4 °C without exposure to light for 2 h. After incubation, cells were washed once in TELi buffer prior to measurements. Nonspecific binding was determined in the presence of a 1000-fold excess of unlabelled ligand (NTR1 variants: 25 μM neurotensin (8–13) (AnaSpec); NK1R variants: 20 μM substance P (AnaSpec); KOR1 variants: 10 μM dynorphin A (1–11) (GenScript)).

### Flow cytometry and FACS

Cells fluorescently labelled by ligand binding were kept in TELi buffer for measurements. Flow cytometry was performed on a BD FACSCanto II cytometer (BD Biosciences) or on a BD LSRFortessa cell analyzer (BD Biosciences) and FACS was performed on a BD FACSAria III sorter (BD Biosciences). For analytical measurements always 50,000 events were recorded. During FACS, in total 3 × 10^5^ − 5 × 10^5^ of the 0.5 − 1.0% most fluorescent cells were sorted into SDD-Leu^–^ medium for subsequent cultivation at 30 °C for 24 h. For all samples identical acquisition settings were used in order to allow comparative analysis. Data were analyzed with FlowJo vX.0.7.

### Radioligand binding with yeast cells

1 × 10^8^ cells (assuming OD_600_ = 1 corresponds to 1 × 10^7 ^cells/mL) were harvested after expression and treated by consecutive washing first in 1 mL ddH_2_O, then 1 mL SPH1 buffer (1 M sorbitol, 25 mM EDTA, 50 mM DTT, pH 8.0), and finally in 1 mL 1 M sorbitol. Next, cells were resuspended in 0.5 mL SPH2 buffer (1 M sorbitol, 1 mM EDTA, 10 mM potassium citrate tribasic, pH 5.8) and cell wall digestion was performed by addition of 6 U/mL Zymolyase 20T (AMS Biotechnology) followed by incubation at 30 °C for 30 min. Subsequently, cells were incubated at 4 °C for 2 h in 200 μl 50 mM Tris-HCl pH 7.4 (at 4 °C) containing [^3^H]-labelled ligand (NTR1 variants: 20 nM [3,11-tyrosyl-3,5-^3^H(N)]-neurotensin (Perkin Elmer); NK1R variants: 15 nM [leucyl-3,4,5-^3^H(N)]-substance P (Perkin Elmer); KOR1 variants: 15 nM [15,16-^3^H]-diprenorphine (Perkin Elmer)). Nonspecific binding was determined in the presence of a 1000-fold excess of unlabelled ligand (NTR1 variants: 20 μM neurotensin (8–13) (AnaSpec); NK1R variants: 15 μM substance P (AnaSpec); KOR1 variants: 15 μM diprenorphine (Tocris Bioscience)). After incubation, cells were filtered on MultiScreen filter plates (Merck Millipore) with a vacuum manifold, filters were washed four times with cold 50 mM Tris-HCl pH 7.4 (at 4 °C), transferred to Isoplate-96 scintillation plates (Perkin Elmer), dried at 65 °C for 2 h, and 200 μL Optiphase Supermix scintillation cocktail (Perkin Elmer) was added. Counting was performed on a 1450 MicroBeta Plus liquid scintillation counter (Wallac). Measured CPM values were normalized to the number of cells used in the assay and the nonspecific signal was subtracted from the total signal.

### Generation of recombinant baculovirus and *Spodoptera frugiperda* (*Sf*9) expression

Wild-type and evolved GPCR genes were amplified by PCR from yeast expression vector pMS03het and cloned via SLIC^35^,^36^ into a modified MultiBac pFL vector^37^,^38^. The vector designated as pFL_mFLAG_His10_TEV_SLIC contains an expression cassette with an N-terminal melittin signal sequence followed by a FLAG-tag, a deca-histidine-tag, a TEV protease cleavage site, and a SLIC cloning site. *E. coli* DH10 EMBacY cells^39^ were transformed with pFL vectors containing the different receptor genes and the resulting baculovirus genome was isolated.

Recombinant baculovirus was generated by transfecting 8 × 10^5^ *Sf*9 cells in 2 mL of Sf 900 II SFM medium (Life Technologies) using 8 μL Cellfectin II reagent (Life Technologies). After 4 h of incubation in a humidified incubator at 27 °C, the transfection medium was removed and replaced by 2 mL of fresh Sf 900 II SFM medium. V_0_ viral stock was harvested after 5 d at 27 °C and used to generate V_1_ high-titre virus stock (1 × 10^8^ − 1 × 10^9^ viral particles per mL). V_1_ virus stock was then used to generate baculovirus-infected insect cell (BIIC) stocks. Briefly, *Sf*9 cells at a density of 1 × 10^6 ^cells/mL were infected with a multiplicity of infection of 5, incubated for 24 h in suspension, harvested and frozen at −80 °C in aliquots in Sf 900 II SFM medium containing penicillin-streptomycin (Life Technologies) and 10% (v/v) DMSO. For long-term storage, BIIC stocks were kept at −150 °C.

Expression was performed in Sf 900 II SFM medium (Life Technologies) by infection of *Sf*9 cells at a density of 3 × 10^6 ^cells/mL with 100-fold diluted BIIC stocks and cultivation at 27 °C for 4 d. After expression, cells were harvested by centrifugation, washed in cold PBS, frozen in liquid nitrogen and stored at −80 °C until use.

### Radioligand binding with *Sf*9 cells

After expression, 1 × 10^4^ cells were incubated at 4 °C for 2 h in 200 μL binding buffer (50 mM Tris-HCl pH 7.4 (at 4 °C), 1 mM EDTA, 0.1% (w/v) BSA, 40 μg/mL bacitracin) containing [^3^H]-labelled ligand (NTR1 variants: 15 nM [3,11-tyrosyl-3,5-^3^H(N)]-neurotensin (Perkin Elmer); NK1R variants: 15 nM [leucyl-3,4,5-^3^H(N)]-substance P (Perkin Elmer); KOR1 variants: 15 nM [15,16-^3^H]-diprenorphine (Perkin Elmer)). Nonspecific binding was determined in the presence of a 1000-fold excess of unlabelled ligand (NTR1 variants: 10 μM neurotensin (8–13) (AnaSpec); NK1R variants: 15 μM substance P (AnaSpec); KOR1 variants: 15 μM diprenorphine (Tocris Bioscience)). After incubation, cells were filtered on MultiScreen filter plates (Merck Millipore) with a vacuum manifold, filters were washed four times with cold 50 mM Tris-HCl pH 7.4 (at 4 °C), transferred to Isoplate-96 scintillation plates (Perkin Elmer), dried at 65 °C for 2 h, and 200 μL Optiphase Supermix scintillation cocktail (Perkin Elmer) was added. Counting was performed on a 1450 MicroBeta Plus liquid scintillation counter (Wallac). Measured CPM values were normalized to the number of cells used in the assay and the nonspecific signal was subtracted from the total signal.

### Membrane isolation from *Sf*9 cells

All steps were performed at 4 °C. Frozen *Sf*9 cells from 100 mL expression cultures (approx. 3 × 10^6 ^cells/mL) were thawed and swelled in 24 mL hypotonic low-salt (LS) buffer (10 mM HEPES pH 7.5, 20 mM KCl, 10 mM MgCl_2_, cOmplete protease inhibitor EDTA-free tablets (Roche)) for 1 h. Cells were disrupted by repeated homogenization (Dounce homogenizer) and membranes were collected by centrifugation at 180,000 rcf for 30 min. Isolated membranes were washed once by repeated homogenization in 24 mL hypertonic high-salt (HS) buffer (10 mM HEPES pH 7.5, 20 mM KCl, 10 mM MgCl_2_, 1 M NaCl, cOmplete protease inhibitor EDTA-free tablets (Roche)) and again collected by centrifugation at 180,000 rcf for 30 min.

For protein purification, the washed membranes were resuspended in 1.3 mL LS buffer, resulting in a total volume of approximately 1.6 mL. For thermostability measurements and saturation/competition binding experiments, the washed membranes were resuspended in 4 mL membrane freezing (MF) buffer (50 mM Tris-HCl pH 7.4 (at 4 °C), 1 mM EDTA, 20% (w/v) sucrose), frozen in liquid nitrogen and stored at −80 °C until use.

### Thermostability measurements

For each sample, 5 μL of washed and homogenized membranes in MF buffer were incubated at 4 °C for 2 h in 200 μL binding buffer (50 mM Tris-HCl pH 7.4 (at 4 °C), 1 mM EDTA, 0.1% (w/v) BSA) containing [^3^H]-labelled ligand (NTR1 variants: 10 nM [3,11-tyrosyl-3,5-^3^H(N)]-neurotensin (Perkin Elmer); NK1R variants: 10 nM [leucyl-3,4,5-^3^H(N)]-substance P (Perkin Elmer); KOR1 variants: 10 nM [15,16-^3^H]-diprenorphine (Perkin Elmer)). Nonspecific binding was determined in the presence of a 1000-fold excess of unlabelled ligand (NTR1 variants: 10 μM neurotensin (8–13) (AnaSpec); NK1R variants: 10 μM substance P (AnaSpec); KOR1 variants: 10 μM diprenorphine (Tocris Bioscience)). After incubation, the samples were incubated in a PCR cycler at different temperatures (25–65 °C) for 20 min. Subsequently, the samples were filtered on MultiScreen filter plates (Merck Millipore) with a vacuum manifold, filters were washed four times with cold 50 mM Tris-HCl pH 7.4 (at 4 °C), transferred to Isoplate-96 scintillation plates (Perkin Elmer), dried at 65 °C for 2 h, and 200 μL Optiphase Supermix scintillation cocktail (Perkin Elmer) was added. Counting was performed on a 1450 MicroBeta Plus liquid scintillation counter (Wallac). The nonspecific signal was subtracted from the total signal and measured values were normalized to 100% activity at 25 °C. Data were analyzed with GraphPad Prism v6.03 by using non-linear regression.

### Radioligand saturation and competition binding experiments

In order to avoid ligand depletion, washed and homogenized membranes in MF buffer were further diluted in MF buffer (NTR1: 10-fold diluted; NTR1-Y06: 50-fold diluted; NK1R: 10-fold diluted; NK1R-Y09: 40-fold diluted).

For each sample in saturation binding experiments, 2.5 μl of diluted membranes in MF buffer were incubated at 4 °C for 2 h in 200 μL binding buffer (50 mM Tris-HCl pH 7.4 (at 4 °C), 1 mM EDTA, 0.1% (w/v) BSA) containing [^3^H]-labelled ligand at different concentrations ranging from 30–0.02 nM (NTR1 variants: [3,11-Tyrosyl-3,5-^3^H(N)]-neurotensin (Perkin Elmer); NK1R variants: [Leucyl-3,4,5-^3^H(N)]-substance P (Perkin Elmer)). Nonspecific binding was determined in the presence of a 1000-fold excess of unlabelled ligand (NTR1 variants: 30–0.02 μM neurotensin (8–13) (AnaSpec); NK1R variants: 30–0.02 μM substance P (AnaSpec)). After incubation, samples were filtered on MultiScreen filter plates (Merck Millipore) with a vacuum manifold, filters were washed four times with cold 50 mM Tris-HCl pH 7.4 (at 4 °C), transferred to Isoplate-96 scintillation plates (Perkin Elmer), dried at 65 °C for 2 h, and 200 μL Optiphase Supermix scintillation cocktail (Perkin Elmer) was added. Counting was performed on a 1450 MicroBeta Plus liquid scintillation counter (Wallac). The nonspecific signal was subtracted from the total signal and data were normalized to the CPM values measured at saturation.

For competition binding experiments, samples were treated and measured analogously to the saturation binding experiments, with the only difference that the binding buffer contained 2.5 nM [^3^H]-labelled ligand (NTR1 variants: [3,11-Tyrosyl-3,5-^3^H(N)]-neurotensin (Perkin Elmer); NK1R variants: [Leucyl-3,4,5-^3^H(N)]-substance P (Perkin Elmer)) as well as competitor at different concentrations ranging from 2500–0.15 nM including a sample without any competitor (NTR1 variants: SR 142948 (Tocris Bioscience); NK1R variants: CP 99994 (Tocris Biosciences)). Data were analyzed with GraphPad Prism v6.03 by using non-linear regression for saturation and competition binding.

### Signalling assays for NK1R variants ([^35^S]-GTPγS binding assay)

G protein (Gαi_1_β_1_γ_1_) was expressed in *Sf*9 cells using a single baculovirus encoding all three subunits. For purification, the N-terminus of Gβ_1_ contained a 3C-protease-cleavable deca-histidine-tag. *Sf*9 cells grown in Sf 900 II SFM medium (Life Technologies) at 27 °C were infected at a density of 7 × 10^6^ cells/mL with a multiplicity of infection of 5 with the G protein-encoding virus and incubated at 27 °C. 3 d after infection, the cells were harvested by centrifugation at 4 °C, resuspended in 30 mL lysis buffer (50 mM HEPES pH 8.0, 50 mM NaCl, 1 mM MgCl_2_, 10 μM GDP, 5 mM β-mercaptoethanol, cOmplete protease inhibitor EDTA-free tablets (Roche)) and lysed by sonication at 4 °C. The lysate was centrifuged at 500 rcf for 5 min (at 4 °C), and the resulting supernatant was centrifuged at 108,000 rcf for 40 min (at 4 °C) to collect the membranes. The G protein was purified according to a procedure previously described^40^.

Membranes of *Sf*9 cells expressing NK1R variants for [^35^S]-GTPγS binding assays were isolated according to an adapted protocol compared to the procedure described above. All steps were performed at 4 °C. The cells were lysed by incubation in lysis buffer (10 mM Tris-HCl pH 7.4 (at 4 °C), 1 mM EDTA, 5 μg/mL Leupeptin, 0.1 mM Pefabloc SC, 1 μg/mL Pepstatin) for 30 min followed by several passages through a 27-gauge needle. Subsequently, membranes were collected by centrifugation and incubated for 30 min in wash buffer (50 mM Tris-HCl pH 7.4 (at 4 °C), 1 mM EDTA) containing 7 M urea to remove peripherally bound proteins. The urea concentration was then reduced to 3.5 M by adding wash buffer, and the membranes were collected again by centrifugation. Finally, the membranes were washed once with wash buffer, resuspended in wash buffer containing 20% (w/v) sucrose, frozen in liquid nitrogen, and stored at −80 °C until use.

For the [^35^S]-GTPγS binding assays, urea-washed membranes containing 1 nM of GPCR were mixed with 100 nM purified G protein and incubated at 25 °C for 20 min in 50 μL assay buffer (50 mM Tris-HCl pH 7.4 (at 4 °C), 1 mM EDTA, 100 mM NaCl, 1 mM DTT, 3 mM MgSO_4_, 0.3% (w/v) BSA, 2 μM GDP (Sigma-Aldrich), 4 nM [^35^S]-GTPγS (Perkin Elmer)) in the presence and absence of 200 μM substance P (AnaSpec). The reaction was stopped by filtration over MultiScreen_HTS_-HA filter plates (Merck Millipore) and subsequent washing for four times with 50 mM Tris-HCl pH 7.4 (at 4 °C). Subsequently, the filters were transferred to Isoplate-96 scintillation plates (Perkin Elmer) and 200 μL Optiphase Supermix scintillation cocktail (Perkin Elmer) was added. Counting was performed on a 1450 MicroBeta Plus liquid scintillation counter (Wallac). Background counts arising from buffer, GPCR and G protein alone have been taken into account and subtracted. Therefore, given counts represent the GPCR-induced [^35^S]-GTPγS binding to G protein in the presence and absence of agonist.

### Purification of NK1R variants from *Sf*9 expression culture

All steps were performed at 4 °C. To approximately 1.6 mL of washed and resuspended membranes in LS buffer either 8 μM substance P (AnaSpec) or 20 μM CP 99994 (Tocris Bioscience) were added together with 2 mg/mL iodoacetamide (Sigma-Aldrich) and the mixture was incubated for 30 min. For solubilization, 1.5 mL solubilization buffer (10 mM HEPES pH 7.5, 1.9 M NaCl, 10 mM MgCl_2_, 20 mM KCl, cOmplete protease inhibitor EDTA-free tablets (Roche)) were added followed by addition of 350 μL of a mixture of 10% (w/v) n-dodecyl-β-D-maltopyranoside (DDM) (Anatrace) and 2% (w/v) cholesteryl hemisuccinate (CHS) (Sigma-Aldrich). After 2 h of solubilization, non-solubilized material was removed by centrifugation at 228,000 rcf for 30 min. The supernatant was then incubated with 150 μL of washed TALON Superflow resin (GE Healthcare) in the presence of 20 mM imidazole overnight. Protein-bound resin was washed in gravity flow columns with 20 column volumes (CV) each of wash 1 buffer (50 mM HEPES pH 7.5, 800 mM NaCl, 10 mM MgCl_2_, 25 mM imidazole, 10% (v/v) glycerol, 0.1/0.02% (w/v) DDM/CHS, 8 mM ATP, supplemented with 10 μM substance P or 10 μM CP 99994, respectively) and wash 2 buffer (50 mM HEPES pH 7.5, 800 mM NaCl, 40 mM imidazole, 10% (v/v) glycerol, 0.05/0.01% (w/v) DDM/CHS, supplemented with 10 μM substance P or 10 μM CP 99994, respectively). Protein was eluted in 4 CV of elution buffer (30 mM HEPES pH 7.5, 800 mM NaCl, 300 mM imidazole, 10% (v/v) glycerol, 0.05/0.01% (w/v) DDM/CHS, supplemented with 20 μM substance P or 20 μM CP 99994, respectively). Size-exclusion chromatography (SEC) was performed on a ÄKTA Pure FPLC system (GE Healthcare) with a Superdex S200 Increase 10/300 GL column (GE Healthcare) equilibrated with SEC buffer (25 mM HEPES pH 7.5, 800 mM NaCl, 0.05/0.01% (w/v) DDM/CHS, supplemented with 5 μM substance P or 1 μM CP 99994, respectively).

### Quantitative Western blot analysis with non-adapted and adapted yeast strains

After expression of NTR1 variants containing a C-terminal HA-tag in non-adapted and adapted yeast strains, 4 × 10^7^ cells (assuming OD_600_ = 1 corresponds to 1 × 10^7 ^cells/mL) for each sample were centrifuged and whole cell protein extraction was performed according to a previously published protocol^41^. Briefly, samples were centrifuged, medium was aspirated, and pelleted cells were resuspended in 500 μL 2 M lithium acetate for incubation on ice for 5 min. Subsequently, cells were pelleted again, the supernatant was removed, 100 μL of 0.4 M NaOH were added, and samples were incubated again on ice for 5 min. After incubation, the samples were centrifuged, the supernatant was removed, and the cell pellets were resuspended in 200 μl reducing NuPAGE LDS sample buffer (Life Technologies). Samples were incubated at 20 °C for 15 min, centrifuged, and 5 μL of each sample were run on a NuPAGE Novex 4–12% Bis-Tris protein gel (Life Technologies) in NuPAGE MES SDS running buffer (Life Technologies). Wet blotting was performed onto Immobilon-FL membranes (Merck Millipore). Blocking of membranes was performed in 1× Casein blocking buffer (Sigma-Aldrich) in PBS at RT for 20 min. Antibody binding was performed in 1× Casein blocking buffer in PBST (PBS, 0.05% (v/v) Tween-20) at RT for 1 h, and PBST was used for all membrane washing steps.

Antibodies used for protein detection were the primary antibodies rabbit anti-HA (Sigma-Aldrich, H6908) and mouse anti-actin (Abcam, ab8224), and the secondary antibodies goat anti-rabbit conjugated to Alexa Fluor 680 (Life Technologies, A-21076) and donkey anti-mouse conjugated to IRDye800 (Rockland Immunochemicals, 610-732-124). Primary rabbit anti-HA antibody was used at a dilution of 1:5,000, primary mouse anti-actin antibody at a dilution of 1:1,000, and the secondary antibodies (goat anti-rabbit conjugated to Alexa Fluor 680 and donkey anti-mouse conjugated to IRDye800) both at a dilution of 1:10,000.

Image acquisition was performed on an Odyssey system (LI-COR Biosciences) and quantification was performed with Image Studio Lite v3.1.4 (LI-COR Biosciences). Data were normalized to the actin signal intensities and the GPCR signal intensity obtained for wild-type NTR1 expressed in the non-adapted strain.

### Confocal fluorescence microscopy with non-adapted and adapted yeast strains

After expression of NTR1 variants containing a C-terminal fusion to mCherry in non-adapted and adapted yeast strains, cells were permeabilized and binding of fluorescent neurotensin was performed as described above. After washing, cells were transferred into Nunc Lab-Tek II chambered coverglasses (Thermo Scientific) and confocal microscopy was performed on a Leica TCS SP5 microscope (Leica Microsystems). For all samples magnification was 630-fold and identical acquisition settings were used in order to allow comparative analysis.

## Additional Information

**How to cite this article**: Schütz, M. *et al*. Directed evolution of G protein-coupled receptors in yeast for higher functional production in eukaryotic expression hosts. *Sci. Rep*. **6**, 21508; doi: 10.1038/srep21508 (2016).

## Supplementary Material

Supplementary Information

## Figures and Tables

**Table 1:** Properties of GPCR variants.

GPCR	Expression levels in *Sf*9 cells		Thermostability		Ligand affinity	
	Receptors percell	Increase comparedto wild-type	T_m_ [ °C]	ΔT_m_ comparedto wild-type [ °C]	Ligand	K_d_^app^ [nM]
NTR1	1.2 × 10^6^	–	43.9 ± 0.3	–	Neurotensin	3.0 ± 0.4
					SR 142948	1.2 ± 1.1
NTR1-Y06	5.5 × 10^6^	~5-fold	47.5 ± 0.5	3.6 ± 0.8	Neurotensin	3.0 ± 0.3
					SR 142948	2.3 ± 1.1
NK1R	0.9 × 10^6^	–	41.8 ± 0.5	–	Substance P	9.4 ± 1.6
					CP 99994	1.0 ± 1.1
NK1R-Y09	4.0 × 10^6^	~4-fold	47.2 ± 1.1	5.4 ± 1.6	Substance P	9.2 ± 0.7
					CP 99994	1.2 ± 1.1
KOR1	0.2 × 10^6^	–	39.5 ± 0.5	–	Dynorphin A	n.d.
					Diprenorphine	n.d.
KOR1-Y05	5.2 × 10^6^	~26-fold	51.9 ± 1.2	12.4 ± 1.7	Dynorphin A	n.d.
					Diprenorphine	n.d.
