# Supplementary material for: Directed evolution of G protein-coupled receptors in yeast for higher functional production in eukaryotic expression hosts
Source: Sci Rep. 2016 Feb 25;6:21508. doi: 10.1038/srep21508 (PMC4766470; doi:10.1038/srep21508)
Supplement: Supplementary Information [file srep21508-s1.pdf]

Supplementary Information

Directed evolution of G protein-coupled receptors in yeast for higher functional production in eukaryotic expression hosts

Marco Schütz[1], Jendrik Schöppe, Erik Sedlák[2], Matthias Hillenbrand, Gabriela Nagy-Davidescu, Janosch Ehrenmann, Christoph Klenk, Pascal Egloff[3], Lutz Kummer[1], and Andreas Plückthun[4]

Department of Biochemistry, University of Zurich, Winterthurerstrasse 190, CH-8057 Zurich, Switzerland

[1]Present address: G7 Therapeutics AG, Grabenstrasse 11a, CH-8952 Schlieren, Switzerland.

[2]Present address: Centre for Interdisciplinary Biosciences and Department of Biochemistry, Pavol Jozef Šafárik University in Košice, 040 01 Košice, Slovakia.

[3]Present address: Institute of Medical Microbiology, University of Zurich, Gloriastrasse 30/32, CH-8006 Zurich, Switzerland.

[4]To whom correspondence should be addressed: Andreas Plückthun; Department of Biochemistry, University of Zurich, Winterthurerstrasse 190, CH-8057 Zurich, Switzerland; Tel.: +41 44 635 55 70; Fax: +41 44 635 57 12; E-mail: plueckthun@bioc.uzh.ch.

a

Position	N	H2		H4	H5					IL3			H6	H7				H8	C			
		2.44	2.50	4.49	5.38	5.49	5.58	5.62	5.69				6.45	7.40	7.42	7.47	7.54	8.53				
	49	107	113	193	237	248	257	261	268	280	284	292	318	356	358	363	370	379	386	392	398	
**NTR1**	**A**	**G**	**D**	**I**	**V**	**F**	**N**	**A**	**V**	**V**	**N**	**N**	**V**	**A**	**F**	**A**	**N**	**V**	**C**	**R**	**R**	**Total mutations**
NTR1-Y01*	P		N					S						S								4
NTR1-Y02**	P		N			L		S		A				S								6
NTR1-Y03**	P	C	N					S						S								5
NTR1-Y04**	P		N					S						S							W	5
NTR1-Y05**	P		N					S						S					G	C		6
NTR1-Y06**	P		N				S	S			D			T	L	T	I			C		10
NTR1-Y07**	P		N	L	A			S	G			D	A	T	L		I	A		C		13

Isolated from library: * NTR1 1.5

** NTR1 2.5

b

Position	H1	IL1	EL1	H3		IL2	EL2	H5			H6	EL3		H7		C					
	1.40			3.36	3.49			5.59	5.64	5.66	6.51			7.34	7.47						
	40	62	95	116	129	138	181	217	222	224	264	274	278	286	299	322	331	335	336	337	
**NK1R**	**T**	**R**	**H**	**V**	**D**	**L**	**M**	**T**	**T**	**W**	**F**	**N**	**Y**	**V**	**M**	**C**	**Y**	**E**	**M**	**K**	**Total mutations**
NK1R-Y01*								S						A						*	3
NK1R-Y02*												Y								*	2
NK1R-Y03*																	*				1
NK1R-Y04*				I			K			R									*		4
NK1R-Y05**				I			K			R						W		K	*		6
NK1R-Y06**	A			I			K			R						W		K	*		7
NK1R-Y07**				I			K									W		K	*		5
NK1R-Y08*	A	S									Y				T					*	5
NK1R-Y09**			R			H	K			R			F			W		K	*		8
NK1R-Y10**					V		K		R		Y					W		K	*		7

Isolated from library: * NK1R 1.5

** NK1R 2.5

c

Position	N			H1	H2				H3				EL2	H6	EL3	H7	C		
				1.28	2.45	2.50	2.55	2.63	3.31	3.35	3.47	3.50		6.42		7.44			
	8	10	24	55	100	105	110	118	137	141	153	156	203	281	301	321	353	377	
**KOR1**	**F**	**G**	**P**	**S**	**N**	**D**	**T**	**V**	**I**	**N**	**S**	**R**	**E**	**A**	**S**	**T**	**E**	**N**	**Total mutations**
KOR1-Y01*							A	D		S	C					I	K		6
KOR1-Y02**		R			S	G		D		S	C				G	I	K		9
KOR1-Y03**					S	G		D		S	C					I	K		7
KOR1-Y04**			R		S	G		D		S	C					I	K		8
KOR1-Y05**					S	G		D		S	C			V		I	K		8
KOR1-Y06**					S	G		D		S	C	H	D			I	K		9
KOR1-Y07**	I			P	S	G		D	T	S	C					I	K	D	11

Isolated from library: * KOR1 1.5

** KOR1 2.5

d

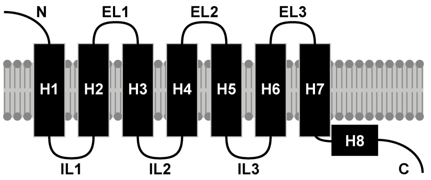

Supplementary Figure 1 | Overview of the most highly enriched clones obtained with SaBRE. Mutations of each clone are indicated and the corresponding wild-type amino acids are given on top of the mutation data. The positions of the mutations are indicated by structural regions, Ballesteros-Weinstein numbering, and sequential amino acid numbering. (**a**) NTR1 variants. (**b**) NK1R variants. (**c**) KOR1 variants. (**d**) Scheme of GPCR topology, depicting the different structural regions harbouring mutations.

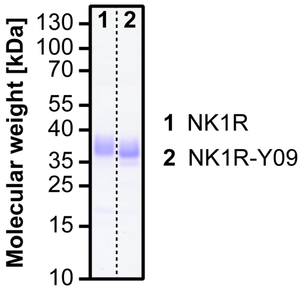

Supplementary Figure 2 | SDS-PAGE analysis of purified wild-type NK1R and NK1R-Y09. Equal amounts of total protein were loaded in each lane. After purification, pure protein is obtained for wild-type NK1R (lane 1) and NK1R-Y09 (lane 2) with little impurities at low molecular weight mainly observed for the wild-type receptor. NK1R runs at lower molecular weight than expected (46 kDa) and the band is less defined than the one obtained for NK1R-Y09, which runs at the expected molecular weight (39 kDa).

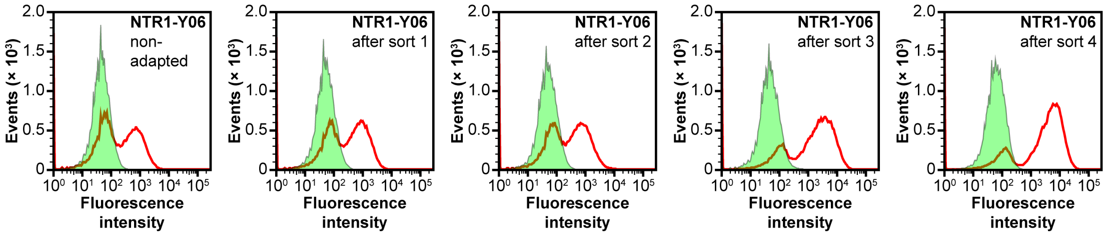

Supplementary Figure 3 | Expression profiles of NTR1-Y06 after individual sorts during repetitive phenotypic selections with FACS for adaptation. Histogram plots of fluorescent ligand binding flow cytometry data with total signal (red curves) and nonspecific signal (green, tinted) are shown. In the course of the phenotypic selection, in which the cells with the highest NTR1-Y06 expression were sorted (gating of the top 1% of the most fluorescent cells), the population of cells not expressing any functional receptor at the surface gradually decreases. At the same time, the specific signal of the expressing cells shifts to higher levels during the selection, indicating an increase of functional receptors at the surface of these cells. Note that the expression profiles did not significantly change after the first two sorts, meaning that two sorts were required to induce adaptation. After the third sort, a clear decrease of the subpopulation with no surface expression of active NTR1-Y06 as well as a shift of the specific signal within the subpopulation which shows surface expression is observed. This trend is continued in subsequent sorts, as seen in the expression profile after the fourth sort.

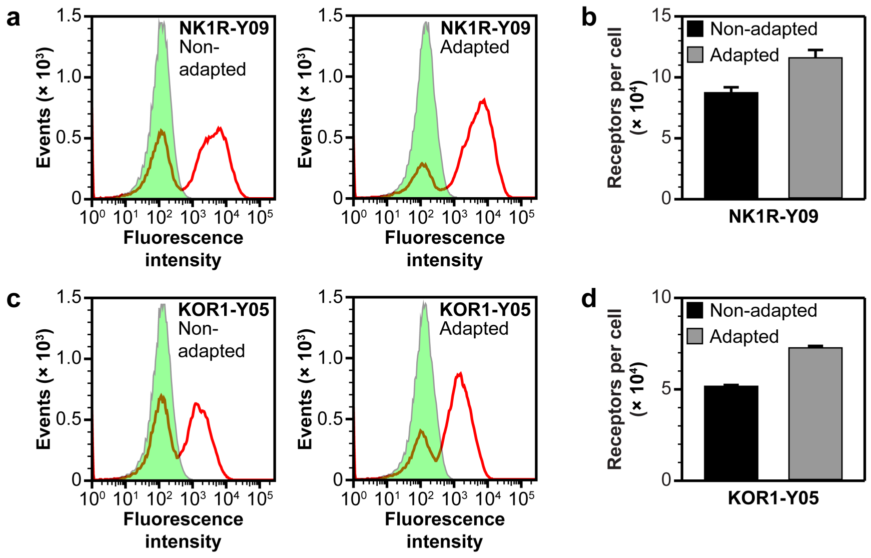

Supplementary Figure 4 | Functional expression of NK1R-Y09 and KOR1-Y05 in non-adapted and adapted yeast strains. (**a**, **c**) Histogram plots of fluorescent ligand binding flow cytometry data with total signal (red curves) and nonspecific signal (green, tinted) are shown. While the specific signals of cells expressing receptor at the surface slightly increase in the adapted compared to the non-adapted strain, a significant decrease of the subpopulation with no surface expression of active GPCR is observed (note the decrease of the left peaks of the total signal double peaks in the adapted compared to the non-adapted strain). (**b**, **d**) Measurement of average total functional GPCRs produced per cell by radioligand binding. Compared to the non-adapted strains, the average functional expression levels of both evolved receptor variants are increased in the adapted strains, which can be explained by the decrease of the fraction of cells not expressing active receptor at the surface, as observed in the flow cytometry experiments. Error bars indicate standard deviations from triplicates.

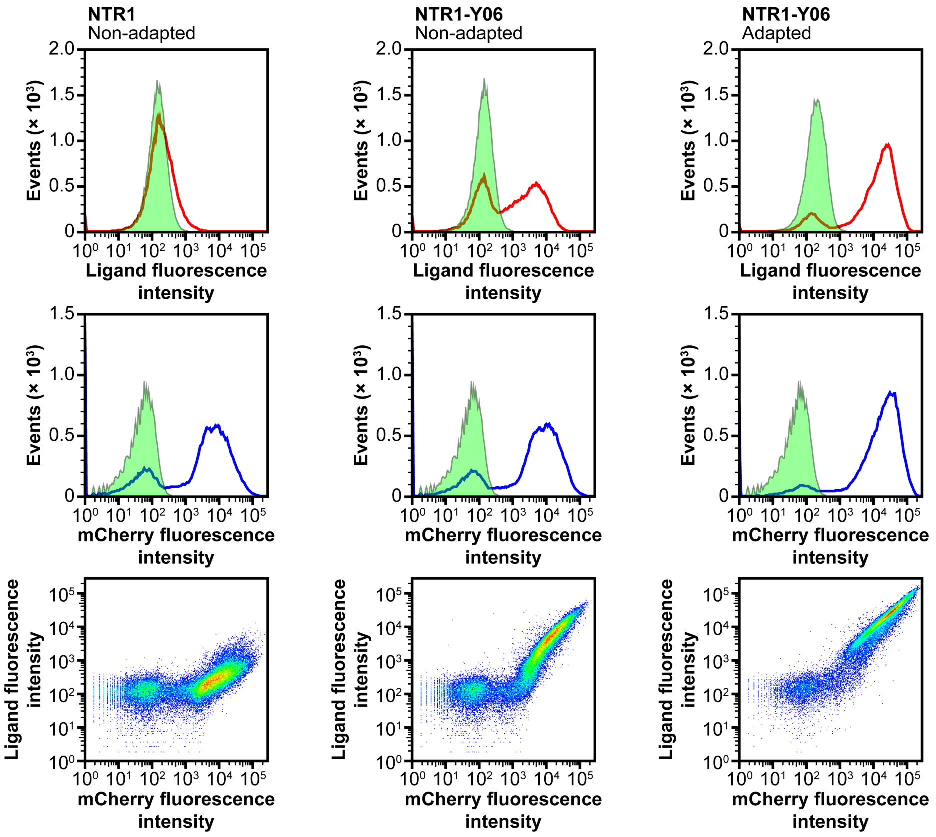

Supplementary Figure 5 | Flow cytometry analysis of non-adapted and adapted yeast strains expressing NTR1 variants with a C-terminal fusion to mCherry. Compared are wild-type NTR1 expressed in the non-adapted strain (left panel), NTR1-Y06 expressed in the non-adapted strain (middle panel), and NTR1-Y06 expressed in the adapted strain (right panel). In the histogram plots of fluorescent ligand binding experiments (top row), the surface expression of active receptors are compared with the total signal (red curves) and nonspecific signal (green, tinted). NTR1 shows little active receptor at the surface, reflected by a low specific signal, and most of the cells do not show any surface expression of NTR1 at all. For expression of NTR1-Y06 in the non-adapted strain, the specific signal is significantly increased compared to the expression of wild-type receptor. Nevertheless, still a significant fraction of cells do not produce active receptor at the surface. For expression of NTR1-Y06 in the adapted strain, the specific signal is further increased, corresponding to more active receptor molecules at the surface compared to expression of NTR1-Y06 in the non-adapted strain. Furthermore, the fraction of cells which show no surface expression of active receptor is significantly decreased (note the decrease of the left peak of the total signal double peak in the adapted compared to the non-adapted strain). In the histogram plots for the detection of mCherry (middle row), the total receptor produced is quantified. Blue curves depict the mCherry signal, whereas autofluorescence of cells expressing NTR1-Y06 without a mCherry fusion, but also incubated with fluorescent ligand, represents the background (green, tinted). For expression of NTR1 and NTR1-Y06 in the non-adapted strains, very similar mCherry signal profiles are obtained. Since for expression of NTR1 in fluorescent ligand binding

experiments only very little active receptor is detected at the surface, the strong mCherry signal indicates that most of the NTR1 receptors must be intracellularly retained. For expression of NTR1-Y06 in the adapted strain, a stronger signal for mCherry is detected. Interestingly, all strains show a fraction of cells showing no expression of mCherry at all, corresponding to a non-expressing subpopulation of cells that may have lost the expression plasmid (note the left peak of the mCherry signal double peak for all three strains). However, for expression of NTR1-Y06 in the adapted strain, this non-expressing fraction of cells is decreased. Note that in fluorescent ligand binding analysis by flow cytometry, this subpopulation of cells cannot be discriminated from cells expressing exclusively intracellularly retained receptors, and together these two subpopulations are detected as the cells which show no active receptor at the surface. In the correlation analysis (bottom row), the mCherry signal (total receptor produced) is compared to the signal obtained with fluorescent ligand binding (functional receptor at the surface). For expression of wild-type NTR1 in the non-adapted strain, functional receptor at the surface does not correlate will with total receptor produced. This correlation is better for NTR1-Y06 expressed in the non-adapted strain, and if NTR1-Y06 is expressed in the adapted strain, functional receptor at the surface correlates well with total receptor produced.

In summary, these data suggest that host adaptation leads to the overall higher average functional GPCR expression due to three effects, namely an increase of functional expression at the surface, a decrease of the fraction of cells exclusively expressing intracellularly retained and mostly inactive receptor, and a decrease of the non-expressing cell subpopulation.
